# Usefulness of Proximal Coronary Wave Speed for Wave Intensity Analysis in Diseased Coronary Vessels

**DOI:** 10.3389/fcvm.2020.00133

**Published:** 2020-08-07

**Authors:** Lorena Casadonte, Jan Baan, Jan J. Piek, Maria Siebes

**Affiliations:** ^1^Department of Biomedical Engineering and Physics, Amsterdam UMC, Amsterdam Cardiovascular Sciences, University of Amsterdam, Amsterdam, Netherlands; ^2^Department of Cardiology, Amsterdam UMC, Amsterdam Cardiovascular Sciences, University of Amsterdam, Amsterdam, Netherlands; ^3^Department of Translational Physiology, Amsterdam UMC, Amsterdam Cardiovascular Sciences, University of Amsterdam, Amsterdam, Netherlands

**Keywords:** wave intensity analysis, wave speed, coronary artery disease, hemodynamics, percutaneous coronary intervention

## Abstract

**Background:** Wave speed is needed to separate net wave intensity into forward and backward traveling components. However, wave speed in diseased coronary arteries cannot be assessed from hemodynamic measurements obtained distal to a stenosis. Wave speed inherently depends on arterial wall properties which should be similar proximal and distal to a stenosis. Our hypothesis is that proximal wave speed can be used to separate net wave intensity obtained distal to a stenosis.

**Methods:** We assessed coronary wave speed using the sum-of-squares single-point technique (SPc) based on simultaneous intracoronary pressure and flow velocity measurements in human coronary arteries. SPc at resting flow was determined in diseased coronary vessels of 12 patients both proximal and distal to the stenosis. In seven of these vessels, distal measurements were additionally obtained after revascularization by stent placement. SPc was also assessed at two axial locations in 14 reference vessels without a stenosis.

**Results:** (1) No difference in SPc was present between proximal and distal locations in the reference vessels. (2) In diseased vessels with a focal stenosis, SPc at the distal location was paradoxically larger than SPc proximal to the stenosis (28.4 ± 3.7 m/s vs. 18.3 ± 1.8 m/s, *p* < 0.02), despite the lower distending pressure downstream of the stenosis. The corresponding separated wave energy tended to be underestimated when derived from SPc at the distal compared with the proximal location. (3) After successful revascularization, SPc at the distal location no longer differed from SPc at the proximal location prior to revascularization (21.9 ± 2.0 m/s vs. 20.8 ± 1.9 m/s, *p* = 0.48). Accordingly, no significant difference in separated wave energy was observed for forward or backward waves.

**Conclusion:** In diseased coronary vessels, SPc assessed from distal hemodynamic signals is erroneously elevated. Our findings suggest that proximal wave speed can be used to separate wave intensity profiles obtained downstream of a stenosis. This approach may extend the application of wave intensity analysis to diseased coronary vessels.

## Introduction

Wave intensity analysis has emerged as a powerful time-domain method to investigate the dynamic interactions between the contracting myocardium and coronary blood flow. Coronary wave intensity distinguishes between concurrently generated upstream and downstream contributions on coronary hemodynamic waveforms and is characterized by forward (aortic) and backward-traveling (microcirculatory) waves (1–3). Compression waves are associated with an increase in pressure, e.g., by propulsion, while expansion waves reflect a decrease in pressure, e.g., by suction.

Wave speed is fundamental to split net wave intensity into separate forward and backward traveling components (4). Local coronary wave speed in normal coronary vessels can be assessed from simultaneously acquired pressure and flow velocity signals (5). The validity of this approach, however, turned out to be compromised under conditions that frequently occur in the clinical setting, such as hyperemia by microvascular dilation or downstream of a stenosis in diseased coronary vessels (6–8).

In patients, wave intensity analysis has been applied to study changes in coronary-cardiac interaction through interventions ranging from pacing and exercise to alterations in left ventricular mechanics by Valsalva maneuver, aortic valve replacement, or left ventricular stunning (9–14). In all these studies, the investigations involved unobstructed coronary arteries.

According to the Moens-Korteweg equation, arterial pulse wave velocity intrinsically depends on the elastic properties of the vessel wall. It can reasonably be expected that the elastic wall properties are similar in angiographically normal vessel sections proximal and distal to a focal stenosis. We therefore hypothesized that proximal wave speed may be suitable to separate the net wave intensity profile obtained distal to a stenosis. To test this hypothesis, we analyzed hemodynamic signals obtained at different axial locations in diseased and unobstructed coronary arteries in patients.

## Materials and Methods

### Hemodynamic Measurements

The dataset for this study was extracted from existing hemodynamic recordings obtained in patients with stable angina pectoris and a single stenosis in a coronary artery, who were scheduled for elective percutaneous coronary intervention (PCI). Exclusion criteria were age below 18 or over 80 years, left main stenosis, subtotal or serial lesions, severe aortic valve disease or heart failure, hypertrophic cardiomyopathy, prior myocardial infarction <6 weeks before PCI or prior cardiac surgery.

Cardiac catheterization was carried out by standard femoral approach. All anti-anginal medication was continued. Intracoronary nitroglycerin (0.1 mg) was administered in order to relax epicardial vessel tone, and coronary angiograms were recorded for quantification of stenosis severity. Aortic pressure (Pa) was measured via the guiding catheter at the coronary ostium. A dual-sensor equipped guide wire (ComboWire XT, Philips-Volcano, Eindhoven, NL) was advanced into the study vessel to simultaneously acquire intracoronary pressure (Pd) and flow velocity (U) signals (15). Hemodynamic signals and ECG were processed by the instrument console (ComboMap, Philips-Volcano, Eindhoven, NL) and digitized at 200 Hz. Flow velocity signals are acquired at 100 Hz by the instrument console and were up-sampled to 200 Hz during offline readout of the digital record. Physiological signals were obtained both proximal and distal to the stenosis during resting flow condition. Depending on stenosis location, proximal measurements were obtained within 2–3 cm from the ostium and distal measurements approximately in the mid- to last third of a vessel. PCI by stent placement was carried out when clinically indicated, and measurements at the distal location were repeated per operator discretion. Care was taken to place the wire sensors at the same downstream location before and after stent placement. Hemodynamic data at equivalent axial locations were also collected in undiseased reference vessels.

### Data Analysis

Vessel dimensions and stenosis diameter reduction were determined by quantitative angiogram analysis (QC-CMS 5.2, Medis Medical Imaging Systems, Leiden, Netherlands). Pressure and flow velocity signals were extracted from the digital recordings and processed using the in-house developed *StudyManager* software (AMC Amsterdam, Netherlands) to derive cycle-averaged values for all relevant variables.

Wave intensity analysis was carried out using custom software written in Delphi (Embarcadero, Austin, TX). The single-point estimate of coronary wave speed (SPc) was assessed according to the sum-of-squares method (5) as reported previously (6–8). In brief, smoothed derivatives of the coronary Pd and U signals were obtained by applying the Savitzky-Golay filter (16) using a third degree polynomial over 11 datapoints. After correcting for the instrument-inherent delay in Pd, SPc was determined using the sum-of-squares technique as

(1) $$\begin{array}{r} SPc=\frac{1}{\rho }\sqrt{\frac{\sum {dP_{d}}^{2}}{\sum {dU}^{2}}} \end{array}$$

A value of ρ = 1,060 kg/m^3^ was used for blood density. Summations were taken over an integer number of three to eight consecutive cardiac periods during resting flow (8). Net coronary wave intensity was calculated from ensemble-averaged beats. Separated forward and backward components of coronary wave intensity (WI), normalized by the sampling rate (17), were then determined as (4)

(2) $$\begin{array}{r} WI_{\pm }=\pm \frac{1}{4\rho c}{\left(\frac{dP_{d}}{dt}\pm \rho c\frac{dU}{dt}\right)}^{2} \end{array}$$

where c is the wave speed in (m/s).

To assess the effect of wave speed on the separated wave intensity, WI was calculated for SPc derived from signals at proximal as well as distal vessel locations. For the dominant forward (positive WI) and backward traveling (negative WI) waves, the area under the respective curve was integrated to yield the corresponding wave energy (in J m^−2^ s^−2^). Four dominant peaks during the cardiac cycle characterize coronary wave intensity waveform: the forward-traveling compression wave (FCW) in early systole and early diastolic backward-traveling expansion wave (BEW), leading to flow acceleration, and the backward compression wave (BCW) and forward expansion wave (FEW), that cause flow deceleration (1, 3, 9).

### Statistics

All values are expressed as mean ± SEM unless otherwise noted. Comparison between results obtained at proximal and distal locations in the same vessel were performed by paired Student's *t*-test (SPSS v.20, IBM, Armonk, NY). Unpaired Student's *t*-test assuming equal variance was used to compare data in reference vessels with those in diseased vessels after PCI. Agreements were assessed with Bland-Altman analysis. A two-sided probability value of *p* < 0.05 was considered significant.

## Results

Simultaneous pressure and flow velocity signals of sufficient quality at rest conditions were collected in 12 patients both upstream and downstream of the stenosis. The patient characteristics are shown in Table 1. Subjects were predominantly male with a mean age of 56 ± 3 years. Coronary artery stenoses were of intermediate severity ranging from 28% (min) to 69% (max) diameter reduction. In seven of these subjects, intracoronary physiology data at the distal location were additionally acquired after PCI. Measurements at two axial locations were obtained in 14 reference vessels.

**Table 1:** Baseline patient characteristics.

Age (years)	56 ± 3
Male sex	10 (83%)
**Diameter reduction (%)**	
Pre-PCI	40.0 ± 3.7
Post-PCI (*n* = 7)	19.3 ± 3.5
**Coronary risk factors**	
Hypertension	1 (8%)
Smoking history	6 (50%)
**Medication**	
Nitrates	8 (67%)
b-Blockers	8 (67%)
Calcium antagonists	4 (33%)

*Values are reported as mean ± SEM or n (%), as appropriate. PCI, percutaneous coronary intervention*.

### Effect of Location on Coronary Hemodynamics and Wave Speed

Hemodynamic variables (mean ± SEM) are summarized in Table 2 for each measurement location. Aortic pressure was normal, with a small pressure gradient across the stenosis at resting flow. With the stenosis present, flow velocity upstream of the stenosis was higher than downstream (*p* < 0.0002) and the pressure difference to aortic pressure was less than downstream of the stenosis (*p* < 0.005).

**Table 2:** Coronary hemodynamics and wave speed.

	**Stenosis (*n* = 12)**	**Post-PCI^1^ (*n* = 7)**	**Reference (*n* = 14)**
**Proximal location**			
HR (bpm)	64 ± 2	63 ± 3	67 ± 3
Pa (mmHg)	98.0 ± 3.3	102.1 ± 3.6	97.4 ± 3.2
Pd (mmHg)	97.0 ± 3.4	101.1 ± 3.7	95.0 ± 3.3
ΔP (mmHg)	1.0 ± 0.2	0.9 ± 0.2	2.5 ± 0.4
U (cm/s)	20.2 ± 1.7	21.7 ± 1.6	21.2 ± 2.5
SPc (m/s)	18.3 ± 1.8	20.8 ± 1.9	23.4 ± 3.3
**Distal location**			
HR (bpm)	65 ± 2	66 ± 3	69 ± 2
Pa (mmHg)	98.2 ± 3.4	97.4 ± 5.7	99.7 ± 3.8
Pd (mmHg)	91.8 ± 3.0	93.8 ± 4.9	96.7 ± 3.9
ΔP (mmHg)	6.5 ± 1.3^†^	3.6 ± 1.3	3.1 ± 0.6
U (cm/s)	14.7 ± 1.7^†^	18.5 ± 1.6	19.9 ± 1.8
SPc (m/s)	28.4 ± 3.7^*^	21.9 ± 2.0	25.0 ± 2.8

*Values are reported as mean ± SEM. HR, heart rate; Pa, aortic pressure; Pd, distal pressure; ΔP, pressure drop; U, flow velocity; SPc, single-point wave speed*.

1 *Proximal values represent those obtained prior to PCI in this patient subgroup*.

* *p < 0.02*,

† *p < 0.005 compared with proximal location*.

**Figure 1:**
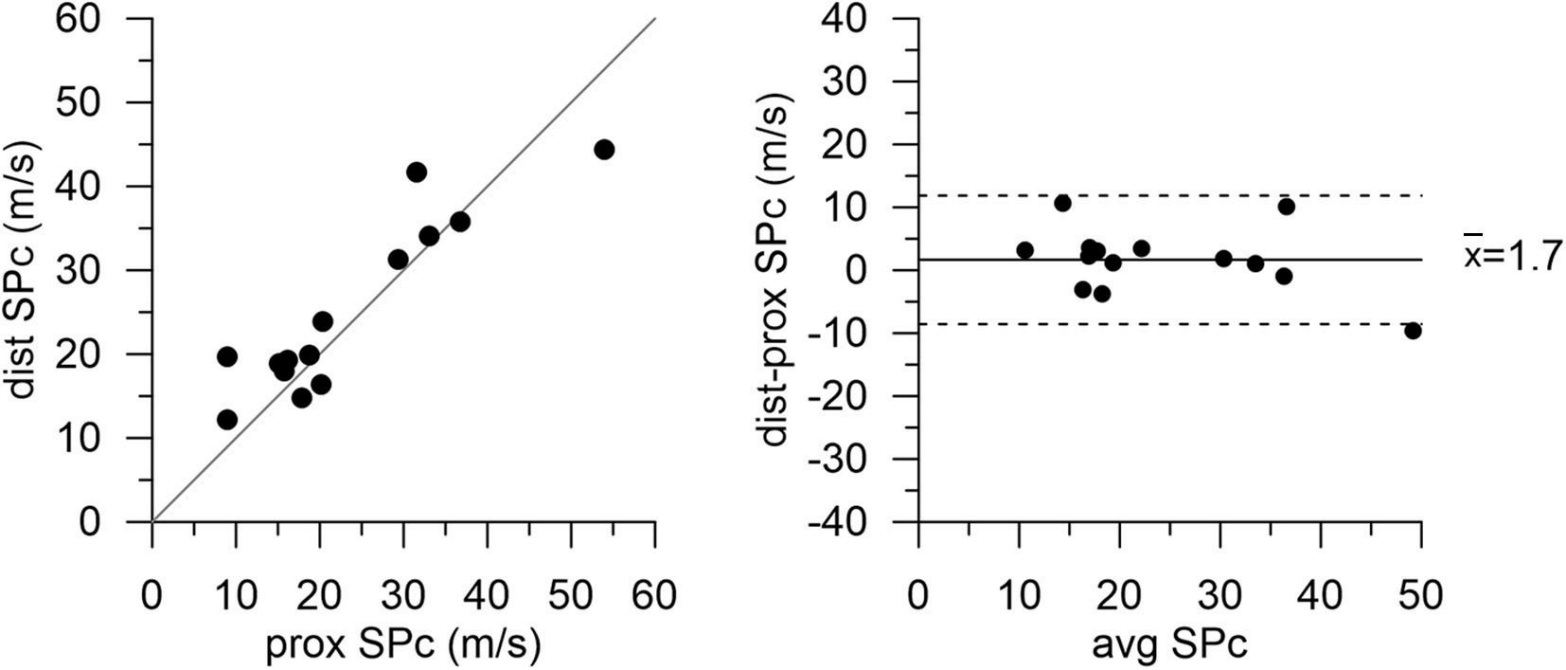
Wave speed (SPc) in normal reference vessels at proximal and distal locations. High correspondence of the data indicates no effect of axial location. Scatterplot with line of identity (left panel) and Bland-Altman analysis (right panel) showing mean bias (solid line) and 95% limits of agreement (dashed lines).

**Figure 2:**
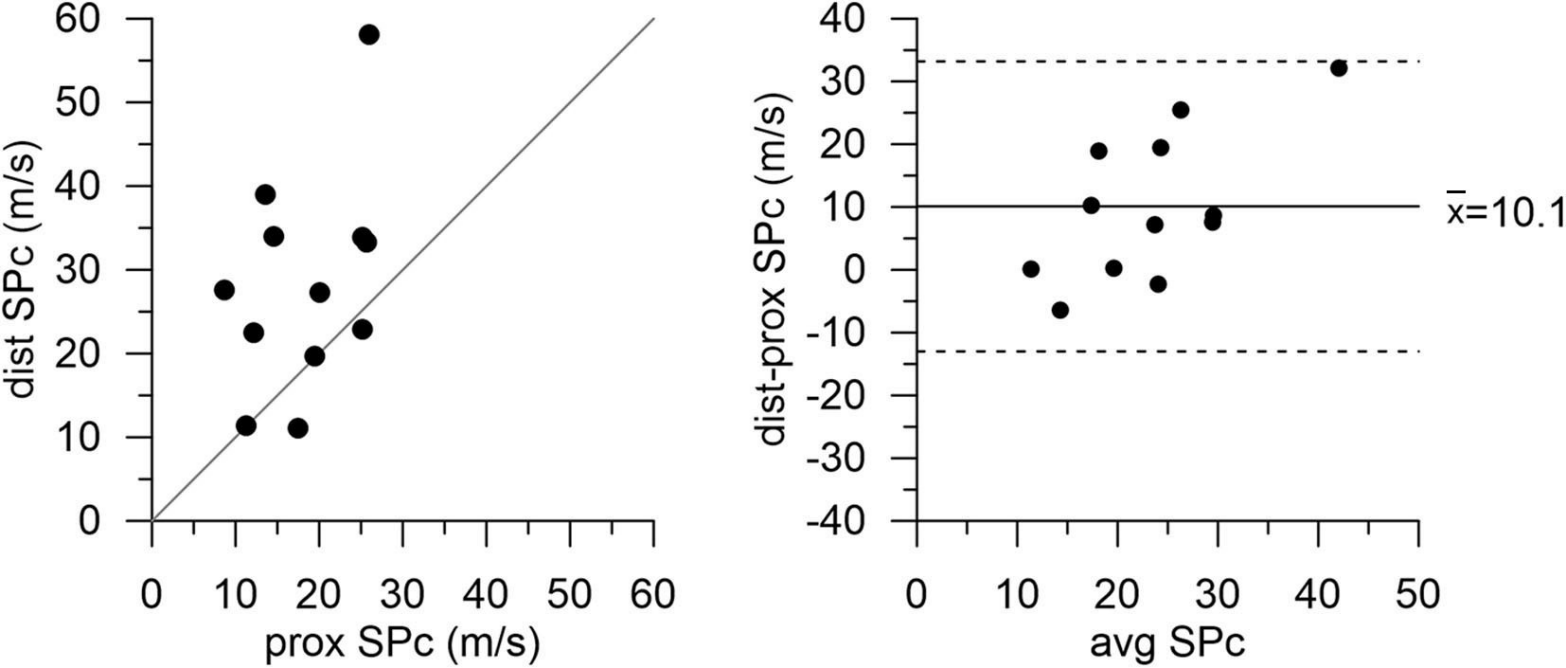
Wave speed (SPc) in diseased coronary vessels proximal and distal to the stenosis. Local SPc downstream of the stenosis is elevated compared with SPc assessed upstream.

**Figure 3:**
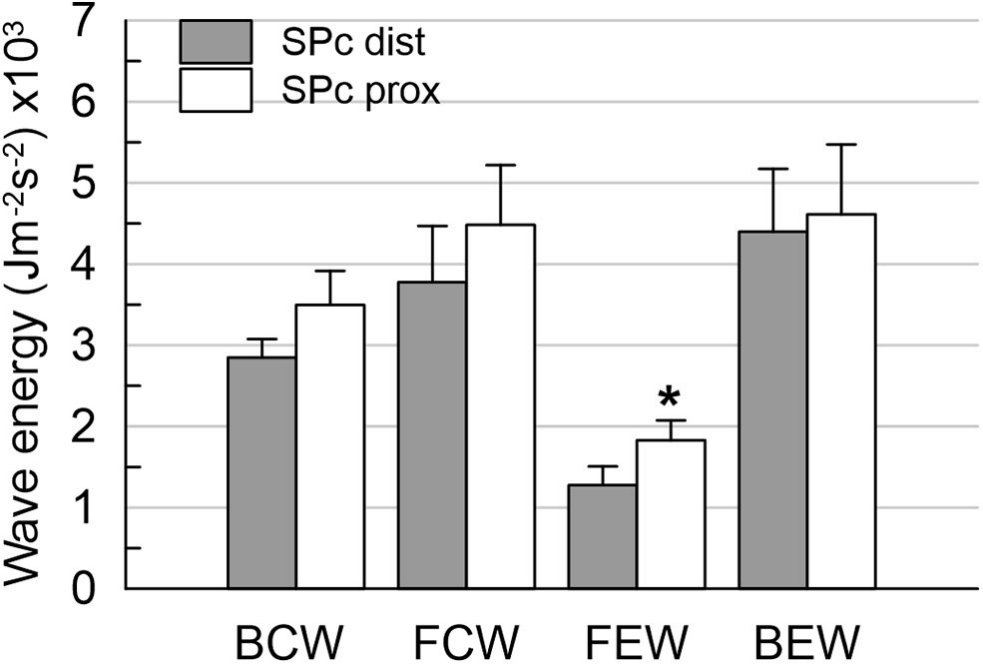
Wave energies downstream of the stenosis obtained from wave intensity profiles separated with local SPc assessed at proximal and distal location. Using distal SPc tends to lower the derived wave intensity. *p < 0.05 compared with distal wave energy.

There was no significant difference in hemodynamics or SPc for different axial locations in the interrogated reference vessels (Figure 1), with an average difference of 1.7 ± 1.4 m/s. In contrast, as illustrated in Figure 2, SPc was significantly larger downstream than upstream of the stenosis (28.4 ± 3.7 m/s vs. 18.3 ± 1.8 m/s, *p* < 0.02). Despite the lower distending pressure downstream of the lesion, SPc at that location was on average 10.1 ± 3.4 m/s higher than upstream. The corresponding separated wave energy tended to be underestimated when calculated using SPc derived at the distal compared with the proximal location (Figure 3).

**Figure 4:**
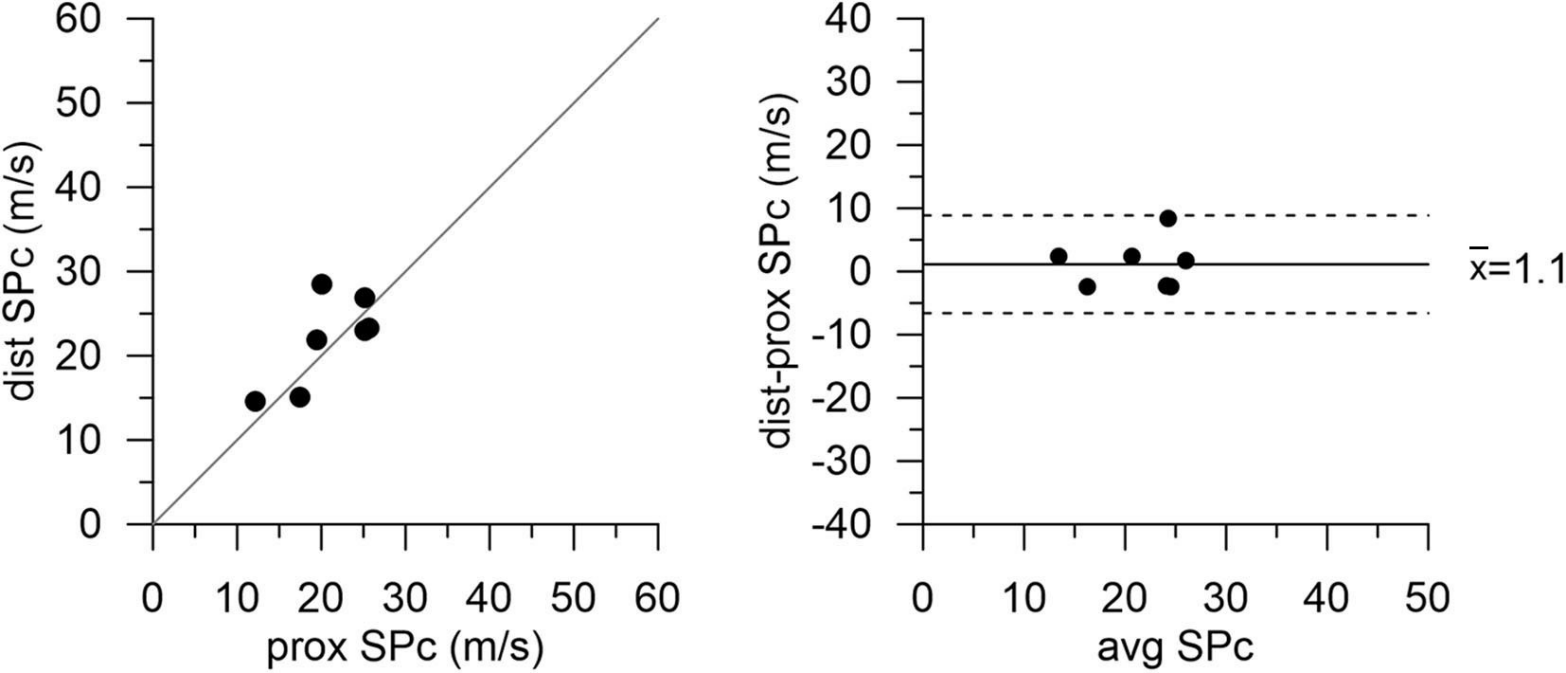
Wave speed (SPc) in diseased vessels at proximal and distal locations after PCI with stent placement. Agreement between measurement locations is close. Note: proximal values are pre-PCI data in this subgroup of patients.

**Figure 5:**
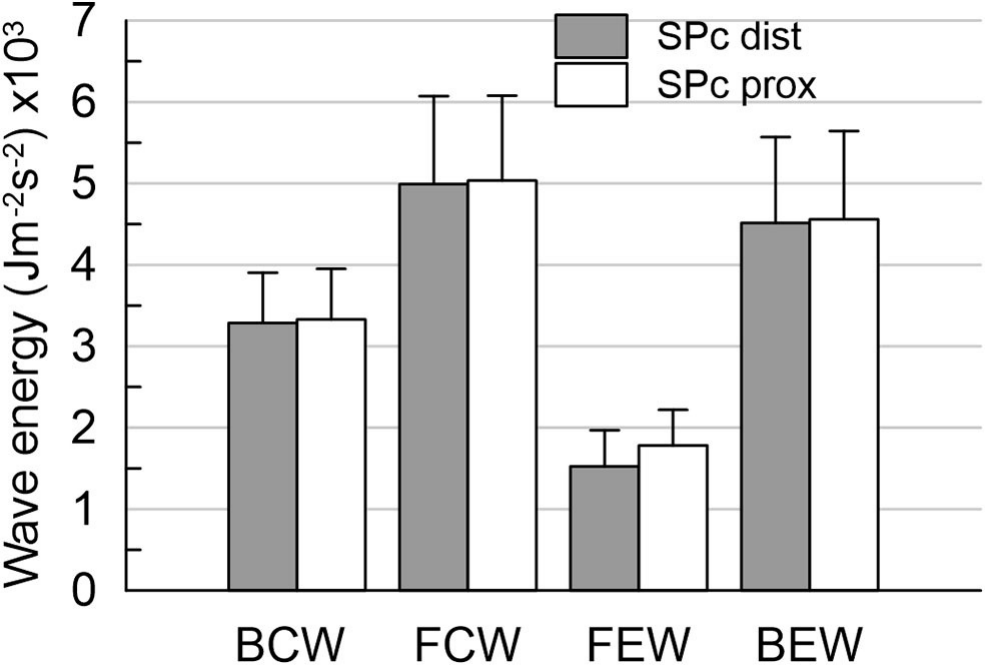
Wave energies at distal location in diseased vessels after PCI. The wave energies are equivalent, whether derived from wave intensity profiles separated with local SPc assessed at proximal or distal locations. Note: proximal values are pre-PCI data in this subset of patients.

After successful revascularization and stent placement, hemodynamics and SPc at the distal location in the treated vessels matched those in the reference vessels. Proximal wave speed did not significantly increase after PCI (*p* = 0.1441). As shown in Figure 4 in the subgroup of patients after PCI, SPc at the distal location no longer differed from proximal SPc in the same vessel assessed prior to revascularization (21.9 ± 2.0 m/s vs. 20.8 ± 1.9 m/s, *p* = 0.48). After PCI, no significant difference in wave energy was observed for either forward or backward separated waves (Figure 5) whether derived using proximal or distal SPc. Bland-Altman analysis confirmed the equivalence with a mean difference of 1.1 ± 1.5 m/s.

## Discussion

In patients with coronary artery disease, this study demonstrated that local coronary wave speed at rest, as derived from the sum-of-squares technique, was higher when assessed in a normal vessel section downstream of a focal stenosis compared with a proximal normal section. That difference was no longer present after the coronary narrowing had been removed by stent placement. Likewise, we did not observe an effect of axial location on coronary wave speed in undiseased reference vessels.

There are only few studies on coronary wave speed in humans. The original sum-of-squares method was first proposed by Davies et al. (5) who tested it in normal coronary vessels without a stenosis. The present findings are consistent with earlier reports by Kolyva et al. (6) and Naranyan et al. (18), which showed that SPc assessed using the sum-of-squares in a vessel segment downstream of a stenosis decreased following PCI. The value of sum-of-squares SPc is governed by the ratio of summed coronary pressure (${\Sigma {\text{dP}}_{\text{d}}}^{2}$) and velocity (ΣdU^2^) oscillations during the cardiac cycle (5). As explained previously (6), in the coronary circulation these systolic-diastolic variations are altered by a stenosis to increase pressure differential-dependent numerator in Equation (1) while decreasing the flow velocity variations in the denominator. SPc assessed by the summed ratio of increased distal pulse pressure and decreased velocity variations downstream of a stenosis is thus at risk to yield artifactually elevated values solely on mathematical grounds. This deviation from expected wave speed behavior despite similar arterial wall properties is enhanced during microvascular vasodilation (6). Rolandi et al. (8) compared SPc with directly measured wave speed in healthy human coronary arteries at rest and during elevated flow after intracoronary administration of adenosine. They found that true coronary wave speed is not altered during hyperemia and hence concluded that the sum-of-squares SPc should be assessed under resting conditions. This inference, however, was limited to healthy vessels and could not be extrapolated to diseased vessels for reasons outlined above.

The current study, using measurements at resting flow, found a difference in proximal vs. distal wave speed only in the presence of a stenosis. Distal SPc was artifactually higher, assuming similar arterial wall properties in the undiseased vessel segments up- and downstream of the focal stenosis. This axial location effect on SPc was not discernible in the same undiseased vessel sections after PCI. A prior study by Siebes et al. (7) demonstrated that simulated overestimation of coronary wave speed in a vessel segment downstream of a stenosis leads to underestimation of the energy contained in the separated forward and backward waves. Our present results clearly show a tendency toward lower wave energy using the higher SPc assessed in a normal vessel segment downstream of the stenosis compared with the integrated wave areas derived using the lower SPc assessed in a proximal vessel segment. Keeping in mind that the relation between wave speed and wave intensity is highly nonlinear, our present findings are consistent with the earlier simulations (7).

Results obtained after revascularization of the stenosis further support our hypothesis that using proximal sum-of-squares SPc likely yields more reliable values for wave intensity downstream of a stenosis, since the difference in SPc that existed before PCI was no longer present. Both proximal and distal SPc values were similar and furthermore matched those obtained in the reference vessels. Interestingly, Harbaoui et al. (19) directly measured coronary wave speed in patients with stable coronary artery disease and found that it was higher in stented vessels than before treatment due to added vessel stiffness imparted by the stent. However, their measurement technique, based on distance traveled and delay in the pressure wave using sequential pressure measurements, covered the entire length of the coronary artery (ca.11 cm) and thus represented global rather than local wave speed. Their invasive technique prevented measurements in healthy vessels for comparison.

The absence of an actual axial location effect on local wave speed in vessel segments of similar stiffness is furthermore corroborated by the comparable results we obtained at different axial locations in the normal reference vessels. This makes sense keeping in mind the predominance of local wall mechanics in determining arterial wave speed under similar hemodynamic conditions.

It should be cautioned that our results pertain to measurements obtained in diseased vessels with intermediate lesions under resting flow conditions, with only a small loss in mean pressure across the stenosis. Pulse wave velocity in arteries depends on distending pressure (20) and extrapolation to hyperemic flow may suffer from mechanical effects associated with the higher pressure loss across the stenosis at elevated flow rates, especially for more severe lesions. In the present study, the difference in pressure gradient before and after PCI was small at the relatively low resting flow values and did not unduly alter vessel wall distensibility.

Future studies in larger patient groups should be directed to investigate the usefulness of proximal SPc assessed at resting flow for wave intensity analysis in diseased vessels during hyperemic conditions.

## Limitations

This observational study includes only a small number of patients. However, this is the first study to compare coronary wave speed at different locations along healthy and diseased coronary arteries. The invasive repeated measurements were well tolerated by the participating subjects, but the duration of the experiments in the catheterization laboratory presented a logistic concern regarding acquisition of invasive measurements at all locations and treatment conditions.

The measurements were obtained in different vessels with different stenosis degrees. Earlier reports have not shown a difference in wave speed between unobstructed left and right coronary arteries (5, 21). Although it cannot be assumed that the vessel wall of a diseased coronary artery is mechanically equivalent to a healthy vessel, we presume that the elastic properties are similar in angiographically normal coronary segments proximal and distal to a focal stenosis or stent. The pertinent proximal-distal comparisons carried out within the same vessel support this assumption. It should be noted that the distance between stenosis and ostium could not be matched due to unavoidable individual differences in stenosis location between patients.

Overall, our mean velocities in patients are consistent with the literature. Mean flow velocity was noticeably lower downstream of the stenosis than upstream. One may argue that this is unusual given the tapering of bifurcating coronary vessels, and that this may explain the higher distal wave speed. Coronary blood flow is well regulated to meet the physiological demand of the perfused downstream myocardial tissue. The velocity distribution in coronary vessels principally depends on the diameter ratio and flow ratio at branch points. The underlying allometric scaling laws (22–24) imply that coronary flow velocity tends to decrease after a branch point. It is not an unusual clinical observation that flow velocity may be diverted away from a stenosed branch and revascularization results in near equalization of proximal and distal mean velocity (25, 26).

A possible influence of reflections at points of geometric or elastic discontinuity on wave speed has not been considered. It has previously been speculated that distal reflection is not a confounding factor for the sum-of-squares method (5), whereas *in vitro* experiments have shown that wave speed is altered within a stenosis or aneurysm (27). The influence of wave reflections on wave intensity in coronary vessels was recently investigated (28). These authors also used a fixed wave speed assessed by the sum-of-squares method. A potential effect of reflection at a stenosis on wave speed in normal sections of coronary vessels remains to be elucidated.

We used the sum-of-squares method to determine local coronary wave speed. Several other single-point techniques have been developed and successfully applied to determine local wave speed in larger human arteries (29). These methods are not applicable in coronary vessels for technical reasons related to the necessary transducers or because theoretical requirements cannot be fulfilled for measurements in the coronary circulation, e.g., finding a linear portion in hemodynamic-loop approaches.

## Conclusion

Our findings suggest that proximal coronary wave speed at resting flow can serve as a suitable surrogate to separate wave intensity profiles obtained downstream of a stenosis. This approach may broaden the scope of wave intensity analysis for the investigation of coronary-cardiac interaction to include diseased vessels. More studies are needed to confirm our early results.

## Data Availability Statement

The datasets used in this study will be made available on reasonable request to the corresponding author.

## Ethics Statement

This study was carried out in accordance with institutional guidelines and the protocol was approved by the local institutional review board (MEC 06/151 #6.17.1610). All subjects gave written informed consent conform the Declaration of Helsinki.

## Author Contributions

MS conceived the study. LC and MS designed the methodology. JB and JP conducted the experiments to collect the intracoronary hemodynamic data. LC performed wave intensity analysis. LC and MS carried out the statistical analysis and wrote the first draft of the manuscript. All authors contributed to the article and approved the submitted version.

## Conflict of Interest

JP serves as a consultant for Philips-Volcano, manufacturer of sensor-equipped guide wires. The remaining authors declare that the research was conducted in the absence of any commercial or financial relationships that could be construed as a potential conflict of interest.
